# Mental disorders and COVID-19 deaths: Clinical, public health, and human rights implications

**DOI:** 10.1371/journal.pmed.1004220

**Published:** 2023-04-11

**Authors:** Shekhar Saxena, Cindy Chwa

**Affiliations:** 1 Department of Global Health and Population, Harvard T.H. Chan School of Public Health, Boston, Massachusetts, United States of America; 2 Department of Social and Behavioral Sciences, Harvard T.H. Chan School of Public Health, Boston, Massachusetts, United States of America

## Abstract

Shekhar Saxena and Cindy Chwa discuss the neglect of care for people living with mental disorders during the pandemic, and highlight relevant implications for policy-makers.

Given the staggering death toll from the Coronavirus Disease 2019 (COVID-19) pandemic, numerous studies have investigated risk factors and care strategies for prevention and treatment. While many observational studies have documented COVID-19-related risk, outcomes, and care cross-sectionally or over a short follow-up period, there is a paucity of longitudinal studies examining COVID-19–associated outcomes across multiple waves of the pandemic, as well as whether the association between preexisting mental disorders and a COVID-19 diagnosis is a result of confounding. With prior longitudinal cohort studies suggesting that preexisting mental disorders are associated with increased mortality from various severe illnesses [1], it is imperative that this research gap is addressed. In an accompanying paper in *PLOS Medicine*, Schwarzinger and colleagues investigated the association between preexisting mental disorders and mortality due to COVID-19 in France, controlling for salvage therapy (higher intensity, rescue treatment given after standard treatments have failed) [2].

Using the French National Hospital Discharge database, Schwarzinger and colleagues assessed mortality among a cohort of adults discharged from acute hospitals with a COVID-19 diagnosis from February 24, 2020 to August 28, 2021 (*N =* 465,750). All participants in this study were followed for 120 days after their first recorded COVID-19 diagnosis, and all-cause mortality data were taken from acute, post-acute, and psychiatric hospitals. Salvage therapy was assessed by first record of intensive care unit admission, oxygenation, respiratory support, or airway pressure therapy during the follow-up period. Four wave periods were considered to investigate the role of COVID-19 caseload surges on prognosis.

Nearly all categories of mental disorder in the study were associated with higher mortality risk and lower salvage rates. Caseload surges in hospitals were associated with a significantly greater increase in excess mortality risk among patients with mental disorders compared to those without. For the majority of the study period, patients without a preexisting mental disorder accessed salvage therapy at higher rates than expected (+4.2%, for a predicted rate of 18.8%) and those with a preexisting mental disorder at lower rates than expected (−4.1%, for a predicted rate of 18.0%). Their finding of a higher mortality rate among people with preexisting mental disorders is in agreement with earlier studies from other countries [3], but this new study adds value through its large sample and longitudinal design, accounting for a substantial number of confounders and analyzing access to salvage therapy, which has been less often reported. Factoring in severe somatic comorbidities is a notable strength, since this can be a significant confounder.

Over the duration of COVID-19 pandemic, various risk factors have been identified for infection, severe illness, and death [4]. Preexisting mental disorders were identified relatively early in the pandemic as a risk factor for COVID-19 infection but remain subject to limited public awareness [5]. Stigmatized narratives surrounding mental health disorders continue to persist, especially the neglect of care for people living with mental disorders. Ultimately, communities that have higher risk of being diagnosed with COVID-19 also tend to have low socioeconomic statuses and lack access to affordable mental healthcare [6]. Large studies like that by Schwarzinger and team can help to address this knowledge gap and can facilitate discussion on the clinical, public health, and human rights implications of these findings.

On the clinical side, present or past mental disorders should be considered as risk factors among people presenting with COVID-19. Healthcare professionals should consider this in their clinical decision-making regarding admissions to inpatient care, prescription of medicines, provision of lifesaving intensive therapies, timing of discharge from inpatient care, and frequency and quality of aftercare. This should be done in a nonstigmatizing manner and in full and open consultation with the patient and their family. Equally importantly, among communities where mental health stigma is more prevalent than others, healthcare practitioners should engage in cultural sensitivity training to understand best ways to treat their patients in accordance with their identities. It should be remembered that the vast majority of persons living with mental disorders are able to understand and make considered judgment and decisions about their care options [7]; it is often that they are not asked.

From a public health perspective, COVID-19 control and healthcare policies need to explicitly recognize people living with mental disorders as a vulnerable group and take this into account in all actions related to public awareness, protection, early detection, and access to treatment. These considerations should also extend to financing of care since people living with mental disorders are more likely to face adverse socioeconomic circumstances and have less access to health insurance, especially in countries where healthcare is not financed by the government [8]. Though the present study focused on patients discharged with a diagnosis of COVID-19, the prevalence and mortality of COVID-19 has also been reported to be high in dementia care facilities [9] and in long-stay psychiatric hospitals [10]. These populations are even more vulnerable, and COVID-19 policy-makers need to direct their attention to them.

Lastly, the study raises substantial human rights issues. People living with mental disorders are subject to stigma and various assaults on their rights [11]; that they are denied provision of COVID-19–related care and lifesaving interventions is an example of such discrimination. This goes against the generally accepted right of highest attainable health [12] and indeed the right to life itself. If the need for a particular intervention (such as lifesaving ventilator support) far exceeds the availability, it is understandable that some criteria will be used to select people who should be given preference, but presence or absence of mental disorders should never be one of these criteria.

As the movement for accountability in healthcare gathers momentum, further studies like this can act as a foundation for more focused and prospective studies to explore the process of decision-making in clinical settings and identify the explicit and implicit biases that exist. This will ensure better accountability in healthcare, especially where stigma and lack of accessible services are key drivers of health inequities.
